# Laser Upcycling of Hemoglobin Protein Biowaste into Engineered Graphene Aerogel Architectures for 3D Supercapacitors

**DOI:** 10.1002/advs.202412588

**Published:** 2024-12-31

**Authors:** Shuichiro Hayashi, Marco Rupp, Jason X. Liu, Joseph W. Stiles, Ankit Das, Amelia Sanchirico, Samuel Moore, Craig B. Arnold

**Affiliations:** ^1^ Princeton Materials Institute Princeton University Princeton NJ 08540 USA; ^2^ Department of Mechanical and Aerospace Engineering Princeton University Princeton NJ 08540 USA; ^3^ Department of Chemical and Biological Engineering Princeton University Princeton NJ 08540 USA; ^4^ Department of Chemistry Princeton University Princeton NJ 08540 USA

**Keywords:** graphene aerogels, laser‐based additive manufacturing, laser‐induced graphitization, powder bed fusion, supercapacitors

## Abstract

Graphene aerogels (GAs) with engineered architectures are a promising material for applications ranging from filtration to energy storage/conversion. However, current preparation approaches involve the combination of multiple intrinsically‐different methodologies to achieve graphene‐synthesis and architecture‐engineering, complicating the entire procedure. Here, a novel approach to prepare GAs with engineered architectures based on the laser‐upcycling of protein biowaste, hemoglobin, is introduced. Laser scanning achieves graphene‐synthesis concurrently with architecture‐engineering through the localized graphitization of hemoglobin along the laser‐scan path, enabling the direct preparation of engineered GAs. The laser‐upcycled GAs are uniquely decorated with fibrous graphitic structures, which significantly improves the surface area. Such structural formation is attributable to the inherent iron content of hemoglobin which leads to the formation of iron‐based nanoparticles that catalyze the formation of nano‐structured graphene. By leveraging the high electrical conductivity and unique structural morphology, the laser‐upcycled GAs are applied as electrodes of symmetrical 3D supercapacitors. The fabricated supercapacitors exhibited a high specific capacitance (≈54.9 F g^−1^) and excellent cycle stability (≈94% retention), attributable to the laser‐engineered architecture facilitating ion diffusion even for thick electrodes. Not only does this study provide a novel approach to prepare GAs with engineered architectures but showcases the potential of laser‐upcycling in preparing advanced functional materials for future devices.

## Graphical abstract

Graphene aerogels (GAs) with engineered architectures are a promising material for various applications. Here, architecture‐engineered GAs are “3D‐printed” by the laser‐upcycling of hemoglobin sourced from blood biowaste. Laser scanning locally graphitizes hemoglobin into low‐density graphene macrostructures along the laser‐scan path, enabling the direct preparation of architecture‐engineered GAs. The laser‐upcycled GAs exhibit eperformances as electrodes of supercapacitors, showcasing the potential of laser‐upcycling in preparing advanced functional materials for future devices.

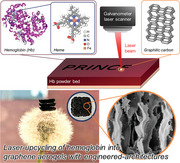

## 1 Introduction

With the constantly depleting reserve of rare‐earth elements, carbon‐based materials have emerged as a sustainable alternative for various fields, as carbon is readily available and exhibits a range of properties depending on its nano‐ to macro‐scaled structure.^[^ ^1^, ^2^ ^]^ Particularly, 2D‐graphene is an attractive nanomaterial owing to its exceptional properties, including high specific surface area, chemical stability, and electrical conductivity.^[^ ^3^ ^]^ By further assembling 2D‐graphene into monolithic 3D networks, generally referred to as graphene aerogels (GAs), highly porous low‐density materials exhibiting advanced mechanical, thermal, chemical, and electrical properties are realized, opening doors to applications in mechanical damping, electromagnetic shielding, water purification, catalysis, and energy storage.^[^ ^4^, ^5^, ^6^, ^7^, ^8^ ^]^

With the spread of 3D printing, the use of additive manufacturing (AM) techniques toward the 3D printing of carbon‐based structures, including GAs, with designed multi‐scaled architectures has been explored.^[^ ^9^, ^10^, ^11^, ^12^, ^13^ ^]^ GAs 3D‐printed with AM techniques exhibited considerably improved performances compared to their solution‐synthesized counterparts owing to the engineered architecture, reinforcing the value of 3D printing.^[^ ^14^, ^15^ ^]^ Architecture‐engineered GAs are mainly achieved using AM techniques based on either extrusion printing of graphene oxide (GO)‐containing inks, or the furnace‐pyrolysis of a 3D‐printed polymeric template. However, these approaches require the combination of multiple intrinsically different methodologies to synthesize the 2D‐graphene units and to form the interconnected hierarchically porous 3D architecture. For example, in the case of extrusion‐based AM, the graphene‐precursor, GO, must be first prepared, typically by solution‐based chemical processes, then carefully formulated into an ink for extrusion printing. Moreover, after 3D printing of the ink, multiple post‐printing processes are required to thermally reduce as well as crosslink the GO into an interconnected graphene network and to chemically etch away residual materials in the ink to finally expose the low‐density 3D architecture. Therefore, despite the multitude of attractive features current AM techniques offer, the inherent complexity and multi‐stepped printing protocol significantly limit scalability. Furthermore, as multiple processes that utilize toxic and unrecyclable chemical materials are involved, current AM techniques lead to harmful secondary waste streams that have severe impacts on the environment.

Laser processing offers the rapid patterning of a wide range of materials along a digitally‐controlled laser‐scan path, analogous to writing with a pen (i.e., laser direct write), and has become an integral part of AM.^[^ ^16^, ^17^ ^]^ The irradiation of a laser beam induces a sequence of physical and chemical reactions at the focal spot leading to the localized subtraction, addition, and/or modification of a target material. Through laser‐induced processes such as ablation, polymerization, melting, reduction, and graphitization, various structures, including polymeric, metallic, ceramic, and carbon‐based, can be rapidly patterned into arbitrary 2D and 3D architectures.^[^ ^18^, ^19^, ^20^, ^21^, ^22^, ^23^ ^]^ In recent years, a laser‐based AM technique that has been attracting a lot of attention is powder bed fusion (PBF).^[^ ^17^, ^24^ ^]^ In this technique, a laser beam is irradiated onto a bed of powdered feedstock material to locally melt and fuse the powder into a continuous macrostructure. By depositing a layer of powder bed and subsequently irradiating a laser beam in succession, 3D macrostructures with arbitrary geometries can be directly printed through a layer‐by‐layer approach. As only the irradiated powder is used for printing and the non‐irradiated powder can be recycled, PBF significantly reduces waste generation and has been discussed to be a highly sustainable and efficient next‐generation AM technique. Moreover, PBF can be applied toward the 3D printing of virtually any material which can be mechanically grounded into powder form and fuses upon laser irradiation, offering high versatility. However, to date, most of the studies regarding PBF have been centered around the 3D printing of metallic and ceramic materials,^[^ ^25^ ^]^ and the application of PBF toward the 3D printing of carbon‐based materials, particularly GAs, has been severely lacking.^[^ ^17^, ^26^ ^]^ As the laser‐assisted 2D patterning of carbon‐based materials has shown immense promise toward the rapid fabrication of planar electronic devices, such as sensors and energy elements,^[^ ^27^, ^28^, ^29^, ^30^, ^31^, ^32^ ^]^ the development of a new laser‐based AM technique capable of 3D printing of carbon‐based materials will further expand the possibilities of laser processing in fabricating devices with advanced functionalities.

Here, we expand on the concept of PBF to prepare engineered GA macrostructures using hemoglobin as the feedstock material, an iron‐containing protein found in red blood cells. Hemoglobin is an abundantly available natural resource that can be sourced from common biowastes of the meat industry (i.e., blood), with millions of tons discarded yearly.^[^ ^33^ ^]^ By the laser irradiation of freeze‐dried hemoglobin powder, low‐density 3D networks of graphene are directly obtained through the localized graphitization of the powder (i.e., laser upcycled) without additional binders, templates, or chemical solutions. By digitally controlling the scan path of the laser beam, GA macrostructures with arbitrary architectures are directly synthesized along the laser scan path. Therefore, the significant distinction from the conventional addition‐based PBF process is that “3D printing” in this approach is based on the combination of both addition and modification laser processes where graphene‐synthesis ensues concurrently with architecture‐engineering, and is not based on the simple fusion of basic units (i.e., feedstock powder). The laser‐upcycled GAs prepared using hemoglobin exhibit low volumetric density and high electrical conductivity, comparable with values reported for conventional solution‐synthesized GAs. Moreover, the inherent iron content of hemoglobin, and the subsequent formation of iron‐based nanoparticles upon laser irradiation, results in the surfaces of the laser‐upcycled GAs to be uniquely decorated with a forest of fibrous graphitic structures. To take advantage of the exceptional structural and material properties of the laser‐upcycled GAs, symmetrical supercapacitors are fabricated using two identical macrostructures bridged by an electrolyte‐infused separator. The supercapacitors fabricated with the laser‐upcycled GAs exhibited exceptional performances, including high specific capacitance, high‐rate capabilities, and excellent cycle stability, despite the large electrode‐thickness. Such device features are attributable to the laser‐engineered architecture facilitating ion diffusion and indicate the applicability of the laser‐upcycled GAs for future energy storage devices.

## 2 Results and Discussion

### 2.1 3D printing Technique Based on Laser‐Upcycling of Hemoglobin

**Figure 1:**
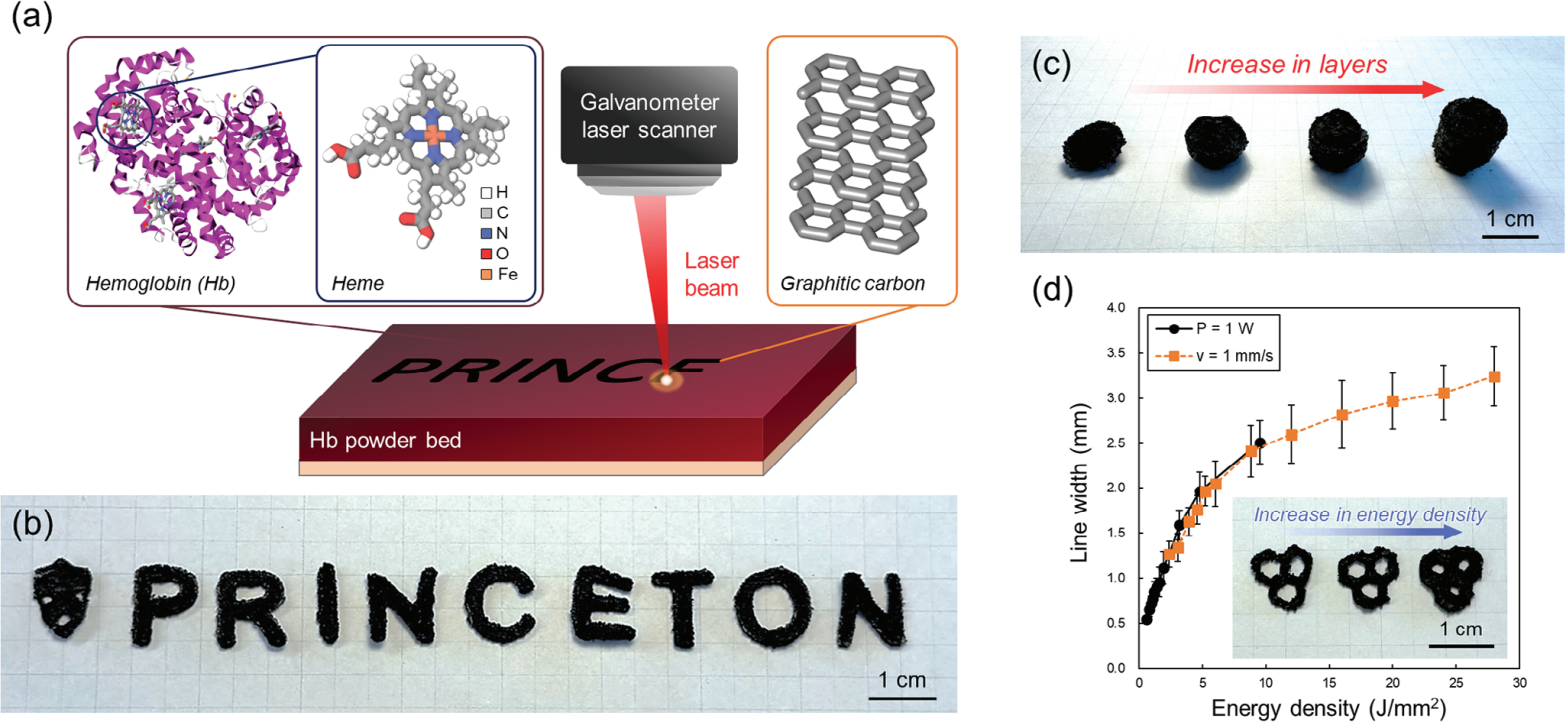
3D laser printing technique using powdered hemoglobin as feedstock. a) Schematic illustration of the laser printing process. Photographs of macrostructures printed by b) a single layer of powder bed, and c) different layers of powder bed (left to right: 1, 2, 3, 4). d) Relationship between the line width of a line structure resulting from a single laser scan (i.e., printing resolution) and the laser energy density. The values and the error bars in d) indicate the mean and standard deviation, respectively (*n*  =  10).

A hemoglobin molecule is an assemblage of four protein subunits, each consisting of one iron‐containing heme group enfolded in a partially coiled polypeptide chain (**Figure** **1**a).^[^ ^34^ ^]^ Analogous to conventional PBF processes, a bed of powdered hemoglobin was deposited onto a working plate, and subsequently irradiated in an inert gas‐flow chamber with a digitally controlled laser beam to print a macrostructure composed of graphene (Figure 1b). The inert gas‐flow chamber assists in preventing unwanted oxidation (i.e., combustion) and removing gaseous by‐products during processing. Moreover, this process can be easily scaled up by simply depositing another layer of hemoglobin powder over the printed macrostructure and subsequently scanning the laser beam (Figure 1c). Through the repetition of these steps, a monolithic macrostructure that can be easily picked up and handled (Figure S1, Supporting Information) with arbitrary 3D geometries such as a cylinder with different heights, can be printed through a layer‐by‐layer approach.

Figure 1d shows the relationship between the width of a line structure printed by a single laser scan and the energy density of the laser beam. The energy density was altered by either changing the laser power or scanning speed while keeping the other parameter constant. Specifically, for the black plots, the laser power was set at 1 W and the scanning speed was altered, while for the orange plots, the scanning speed was set at 1 mm s^−1^ and the laser power was altered. With the increase in energy density, through either a decrease in scanning speed (black plots) or an increase in laser power (orange plots), a sharp increase followed by saturation in line width is observed. As a continuous near‐IR laser beam (≈1060 nm) was used for the process, typical of PBF, the formation mechanism is expected to be through photothermal effects rather than photochemical effects.^[^ ^35^ ^]^ Therefore, the dimensions of the resulting macrostructure will depend on the dimensions of the heat‐affected zone (HAZ) resulting from laser thermalization. With an increase in energy density, the attained peak temperature will increase, resulting in a wider HAZ. However, the growth in dimensions of the HAZ according to an increase in energy density will start to saturate due to diffusion limitations.^[^ ^36^, ^37^ ^]^ The printing resolution (i.e., line width of the structures resulting from a single laser scan) of the current AM technique greatly depends on the dimensions of HAZ. By further locally confining the HAZ through efficient thermalization, for example, by optimizing the laser wavelength, using ultrafast pulsed lasers, and/or implementing advanced scanning schemes,^[^ ^38^, ^39^, ^40^ ^]^ significant improvement in printing resolution (<20 µm) may be achievable in the future. On a different note, it is observed that the black plots are overlayed over the orange plots with a similar trend, suggesting that the printing resolution scales with energy density for the investigated parameter range. Recently we indicated that the laser‐induced formation of carbon‐based structures cannot always be discussed in terms of energy density as the laser power and scanning speed influence the mechanism in different ways.^[^ ^41^ ^]^ Specifically, since the scanning speed determines the resident time of the laser beam (i.e., reaction duration), structural formation can be severely suppressed for high scanning speeds (i.e., 400–1000 mm s^−1^) due to limitations of nucleation kinetics. However, considering the relatively low scanning speeds explored in this study (i.e., 0.25–4.00 mm s^−1^), the influence of nucleation kinetics was presumably negligible, resulting in the similar trend regardless of laser parameter alteration.

### 2.2 Influence of Laser Energy Density on Material Evolution

**Figure 2:**
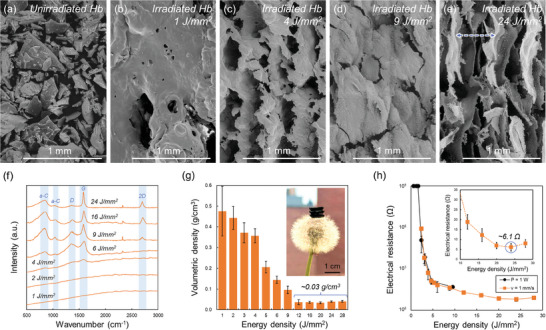
Characterization of macrostructures printed with different energy densities. SEM images of the a) unirradiated powder precursor, and macrostructures printed with an energy density of b) 1, c) 4, d) 9, and e) 24 J mm^−2^. f) Representative Raman spectra obtained from macrostructures printed with different energy densities. Measured g) volumetric densities and h) electrical resistances for macrostructures printed with different energy densities. The values and the error bars in g) and h) indicate the mean and standard deviation, respectively (*n*  =  10). Inset in g) shows a photograph of 3 square macrostructures stacked on top of a dandelion, displaying that the laser‐printed GAs are lightweight.

To characterize the hemoglobin‐derived macrostructures, square macrostructures were printed by raster scanning the laser beam once over a 1 cm by 1 cm area of powder bed (Figure S2, Supporting Information). The hatch spacing (400 µm) was set to be less than the beam diameter (500 µm) so that consecutive raster scans were overlapping and ensured the entire area was irradiated. **Figure** **2**a–e shows scanning electron microscopy (SEM) images of the unirradiated hemoglobin (Figure 2a), and the surface of the macrostructures printed with different energy densities (Figure 2b–e). The laser beam was raster scanned in the vertical direction of the images. Prior to laser irradiation, the hemoglobin powders possess a shard‐like glassy morphology (Figure 2a). Upon irradiation of a laser beam (i.e., 1 J mm^−2^), the powders lose shape and fuse to form a continuous macrostructure (Figure 2b). Considering that the surface of the macrostructure is globular, typical of amorphous polymeric byproducts, and possesses multiple pores and blisters, it is expected that structural formation is initiated by the melting and coalescence of neighboring hemoglobin powders. As the energy density is increased from 1 to 4 and 9 J mm^−2^, a gradual evolution in the surface morphology from globular to planar is observed (Figure 2c and d, respectively). When the energy density is further increased to 24 J mm^−2^, a periodically oriented interconnected network is observed (Figure 2e). Note that the orientation (vertically) and periodicity (indicated with the blue arrow) of the networks observed in Figure 2e correspond to the laser scanning direction (vertically) and hatch spacing (400 µm), respectively. This demonstrates that the current laser technique not only possesses the capability to engineer the macro‐scaled architectures (i.e., geometry) but also the micro‐scaled architectures (i.e., network). Moreover, the cross‐sectional SEM image reveals that the network three‐dimensionally extends to the bottom of the macrostructure and is not a simple surface feature (Figure S3a, Supporting Information).

Figure 2f shows representative Raman spectra obtained from the macrostructures printed with different energy densities. For energy densities of 1 and 2 J mm^−2^, notable peaks cannot be distinguished, and significant autofluorescence background is observed (i.e., sloping baseline), suggesting the existence of a significant amount of polymeric residue.^[^ ^42^ ^]^ As the energy density is increased to 4 and 6 J mm^−2^, the emergence of peaks at ≈830, ≈1030, ≈1360, and ≈1580 cm^−1^ is observed. The first two peaks at ≈830 and ≈1030 cm^−1^ are attributable to sp^3^ carbon vibrational modes of hydrogenated amorphous carbon (a‐C), while the latter two peaks at ≈1350, and ≈1580 cm^−1^ are attributable to in‐plane sp^2^ carbon vibration modes of graphitic carbon commonly referred to as the D and G bands.^[^ ^43^ ^]^ The appearance of such peaks, in addition to the gradual weakening in autofluorescence background, suggests that for such energy densities, the onset of graphitization and the nucleation of graphitic domains ensues. As the energy density is further increased to 9, 16, and 24 J mm^−2^, the emergence of an additional peak at a wavenumber of ≈2700 cm^−1^ is observed. This peak is attributable to the out‐of‐plane second‐order sp^2^ carbon resonance modes of graphitic carbon and is commonly referred to as the 2D band. The emergence of the broad symmetrical 2D band indicates the formation of multilayered and twisted graphene, generalized as turbostratic graphene.^[^ ^44^ ^]^ Moreover, in the case of turbostratic graphene, a narrower and stronger 2D band peak and a smaller intensity ratio between the D and G bands (I_D_/I_G_) suggest a greater dimension in the graphitic domains.^[^ ^43^, ^44^ ^]^ According to this, it can be concluded that with an increase in energy density, the graphitization of hemoglobin progressed, resulting in the gradual expansion of graphitic domains (Figure S4, Supporting Information). Nonetheless, the lingering a‐C peaks even for an energy density of 24 J mm^−2^ indicate that amorphous domains remain even for such high energy densities. The spectral evolution, particularly regarding the peaks attributable to graphitic carbon, suggests that the SEM‐observed structural evolution from globular to planar corresponds to the material transition from amorphous polymeric residue to crystalline graphitic carbon with an increase in energy density. Note that the small peak at a wavenumber of ≈2330 cm^−1^ is attributable to atmospheric nitrogen during analyses, and does not originate from the analyzed material.

Figure 2g shows the measured volumetric densities for the macrostructures printed with different energy densities. With an increase in energy density, the volumetric density of the resulting macrostructure decreased, until it eventually saturated at a volumetric density of ≈0.03 g cm^−3^. Within the particular energy density range where the volumetric density saturated, the mass yield of macrostructure for every 1.0 g of hemoglobin consumed was ≈50% (≈0.5 g), and a substantial difference in mass yield (i.e., the difference in volume) was not observed (Figure S5, Supporting Information). This may be due to competing effects of the top surface of the macrostructure ablating (decrease in mass) and the bottom side of the macrostructure growing (increase in mass). Although the formation of 3D graphitic macrostructures by the laser‐induced graphitization of synthetic thermoplastic polymers (e.g., polyimide) has been previously reported,^[^ ^22^, ^26^, ^45^, ^46^ ^]^ the volumetric densities of the resulting macrostructures were orders of magnitude higher than those reported for GAs. Unlike these previous reports on laser‐induced graphitization, the laser‐printed macrostructure in this study indicated an exceptionally low density, comparable to values for GAs 3D‐printed with GO‐based extrusion printing techniques.^[^ ^10^ ^]^ It has recently been reported that some proteins undergo extensive foaming during furnace‐assisted graphitization, resulting in the formation of ultra‐lightweight GAs.^[^ ^7^ ^]^ This unique foaming behavior of protein precursors may have contributed to the formation of exceptionally low‐density graphitic structures in this study even through laser irradiation.

Figure 2h shows the electrical resistances measured via the two‐probe technique for the macrostructures printed with different energy densities. Similar to Figure 1d, the energy density was altered by either changing the laser power or scanning speed, while keeping the other parameter constant. Probes were directly contacted to the top surface of the 1 cm by 1 cm macrostructures with a probe‐to‐probe distance of ≈8 mm. The thickness of the macrostructures for each energy density is listed in Figure S6 (Supporting Information). While the laser‐printed macrostructures possess a periodically oriented network, electrical conductivity is confirmed whether characterizations are performed parallel or perpendicular to the network (Figure S7, Supporting Information). This is due to the fact that the neighboring sections of the network are not completely isolated and are locally bridged as observed in the cross‐sectional SEM images (Figure S3a, Supporting Information). Nonetheless, as a slightly lower resistance was measured when the probes were oriented parallel to the laser scanning direction, all measurements were conducted with such configuration. For an energy density range of 0.6 to 1.6 J mm^−2^, the printed macrostructure did not exhibit electrical conductivity (>GΩ). As the energy density was increased to 4.6 J mm^−2^, through either a decrease in scanning speed (black plots) or an increase in laser power (orange plots), a sharp decrease in resistance from MΩ to sub‐kΩ orders is observed. As the energy density is further increased, the resistance gradually saturates, exhibiting a minimum value of ≈6.1 Ω for an energy density of 24 J mm^−2^. This value is comparable with leading values reported for porous graphitic material formed by the laser‐induced graphitization of other biomass materials (e.g., wood and leaves).^[^ ^47^, ^48^ ^]^ The initial exponential decrease in resistance with an increase in energy density can be attributed to the formation of a conductive percolation network consisting of turbostratic graphene. As the graphitic domains grow with an increase in energy density (as suggested by Raman spectral evolution in Figure 2f), the number of conductive paths increases, leading to improved electrical conductivity. The saturation in resistance may be due to various factors, including limitations in crystallization kinetics and onset of laser ablation. Note that a further increase in energy density was avoided at this time with the current experimental setup, as it resulted in irreversible damaging to the optical elements (Figure S8, Supporting Information).

### 2.3 Micro‐ and Nano‐Scaled Characterizations of Laser‐Upcycled Graphene Aerogels

**Figure 3:**
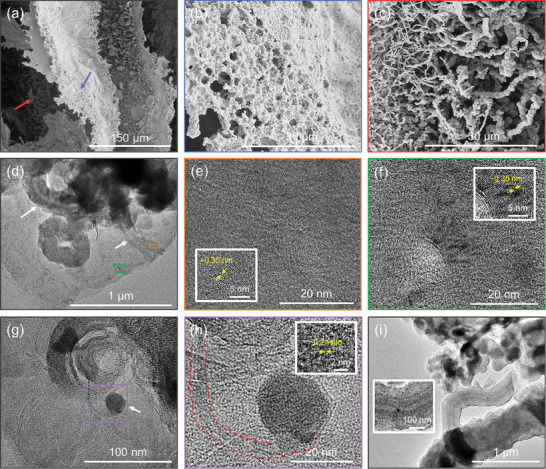
Micro‐ and nano‐scaled features of macrostructures printed with an energy density of 24 J mm^−2^. a) SEM image of the surface of the network wall. b) and c) Higher magnification images of the regions indicated with a blue and red arrow in a), respectively. d) TEM image of nanomaterials formed due to laser irradiation. e) and f) Higher magnification images of the regions indicated with an orange and green rectangle in d), respectively. Insets in both e) and f) show lattice fringes typical of turbostratic graphene. g) TEM image showing the formation of a denser nanoparticle embedded in a graphitic matrix. h) High magnification image of the region indicated with a purple rectangle in g). Inset shows lattice fringes typical of iron‐based nanoparticles. i) TEM image showing the existence of the nanoparticle inside a tubular structure.

As the laser‐printed microstructures exhibiting favorable densities and electrical conductivities are obtained with an energy density of 24 J mm^−2^, the micro‐ and nano‐features of such macrostructures were further characterized (**Figure** **3**). From the higher‐magnification SEM images of the macrostructure surface shown in Figure 2e, two different morphologies of the network wall are observed (Figure 3a). For example, the network wall near the surface (indicated with the blue arrow), is a continuous microstructure possessing multiple micro‐scaled pores (Figure 3b). On the other hand, the network wall deeper within the macrostructure (indicated with the red arrow), is decorated with a dense forest of fibers (Figure 3c). Similar, but thinner fibers are confirmed near the bottom of the network wall as well (Figure S3b,c, Supporting Information), revealing that the entire wall is decorated with such features. Although macroscopically different, the continuous porous microstructure and fibers both seem to result from the aggregation of smaller bulbous subunits. At the current stage, it is unclear whether the continuous microstructure resulted from the coalescence of neighboring fibers, if the fibers sprouted after the formation of continuous porous microstructures, or if they each formed via entirely different mechanisms. Further observations of the fibers via transmission electron microscopy (TEM) suggest that they are hollow, and possess a tubular structure (Figure 3d, indicated with the white arrows). Lattice fringes with a spacing of ≈0.35 nm, corresponding to the (002) plane of turbostratic graphene, are confirmed for both the tubular nanomaterial (Figure 3e) and the surrounding sheet‐like nanomaterial (Figure 3f). In addition to the aforementioned nanomaterials, denser nanoparticles are also observed (Figure 3g, indicated with the white arrow). A different lattice fringe with a spacing of ≈0.24 nm is measured (Figure 3h), which may be attributable to the (112) plane of Fe_3_C.^[^ ^49^ ^]^ This is further supported by the elemental analyses of the denser nanoparticles indicating the evident presence of iron, in addition to carbon (Figure S9, Supporting Information).

One of the characteristics of hemoglobin uniquely different from other proteins is the inherent presence of iron in the molecular structure. Although the concentration of iron in hemoglobin is low, a striking amount of iron is retained after laser irradiation (Figure S10, Supporting Information), sufficient for the nucleation and growth of iron‐based nanoparticles. Such iron‐based nanoparticles, particularly Fe_3_C, can serve as catalysts for nanostructured graphene formation (i.e., iron‐catalyzed graphitization).^[^ ^50^ ^]^ This can occur through either a base‐growth or a tip‐growth mechanism, where graphene nucleates, grows, and exfoliates from the nanoparticle surface, or where the nanoparticle moves through an amorphous carbon medium while leaving a trail of tubular graphene behind, respectively.^[^ ^50^ ^]^ In this case, it is speculated that both mechanisms may be contributing to the formation of nanostructured graphene, as implications of both graphene exfoliation (Figure 3h, dotted red region) and particle movement (Figure 3i) are observed. It is interesting to note that the surfaces of hemoglobin‐derived GAs prepared by furnace‐assisted graphitization were smooth and not decorated with tubular structures (Figure S11, Supporting Information). Moreover, tubular structures were also not observed for laser‐assisted graphitization of polymeric precursors with iron‐rich additives,^[^ ^51^ ^]^ which may suggest that the observed surface decoration is unique to the laser processing of hemoglobin.

### 2.4 Implications of Laser‐Upcycled Graphene Aerogels for Energy Storage

**Figure 4:**
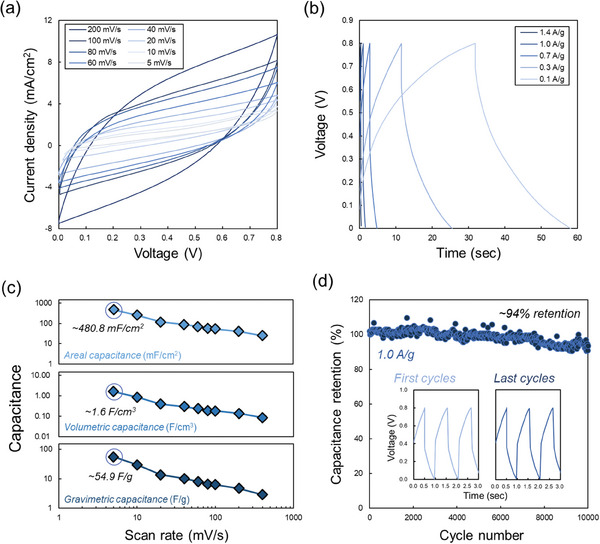
Application of laser‐upcycled GAs for symmetrical supercapacitors. a) Obtained cyclic voltammetry curves for different scan rates. b) Obtained chronopotentiometry curves for different current densities. c) Calculated areal, volumetric, and gravimetric capacitances for different scan rates from CV curves. d) Capacitance stability of the device as a function of the number of cycles at a current density of 1.0 A g^−1^.

Owing to the intrinsically high electrical conductivity and surface area, the laser‐upcycled GAs are hypothesized to have applications as electrodes in electrochemical energy storage devices, such as supercapacitors. A symmetric supercapacitor was fabricated by bridging two square macrostructures (24 J mm^−2^) with a separator infused with an aqueous electrolyte (5 m H_2_SO_4_) (Figure S12, Supporting Information). **Figure** **4**a,b are cyclic voltammetry (CV) and chronopotentiometry (CP) curves obtained from the fabricated supercapacitor for different voltage scan rates and current densities, respectively, after 4 cycles. The semi‐rectangular and semi‐triangular shapes of the CV and CP curves, respectively, indicate evident electric double‐layer capacitance (EDLC) at the interface between the electrode and electrolyte.^[^ ^52^ ^]^ Moreover, EDLC persists for high scan rates and current densities, indicating high‐rate capabilities. For low scan rates, slight undulations in the CV curve are confirmed, suggesting the presence of redox reactions.^[^ ^52^ ^]^ Such redox behavior is commonly observed for supercapacitors with Fe/C‐composites as electrodes and is attributable to the presence of iron‐containing byproducts, such as the crystalline nanoparticles observed via TEM and/or amorphous hemoglobin‐derived polymeric residue.^[^ ^53^, ^54^, ^55^ ^]^ Figure 4c shows the capacitances of the fabricated supercapacitor at different scan rates calculated from the obtained CV curves. The contact electrode area, volume, and mass per electrode were each ≈0.4 cm^2^, ≈0.12 cm^3^, and ≈0.0035 g. For a scan rate of 5 mV s^−1^, the fabricated supercapacitor exhibits a maximum areal capacitance of ≈480.8 mF cm^−2^, volumetric capacitance of ≈1.6 F cm^−3^, and a gravimetric capacitance of ≈54.9 F g^−1^. The current laser‐upcycled GAs prepared using hemoglobin exhibits improved or similar performances compared to other carbon‐based materials used for 3D‐printed supercapacitors, including carbon black (Gravimetric capacitance: ≈25.6 F g^−1^),^[^ ^56^ ^]^ GO (Areal capacitance: ≈153.6 mF cm^−2^),^[^ ^57^ ^]^ laser‐induced graphene foams (Volumetric capacitance: ≈1.5 F cm^−3^),^[^ ^45^ ^]^ graphene/carbon‐nanotube composites (Areal capacitance: ≈639.6 mF cm^−2^),^[^ ^58^ ^]^ and GO‐derived GAs (Gravimetric capacitance: ≈55.5 F g^−1^).^[^ ^9^ ^]^ Moreover, as shown in the Ragone plot, the current supercapacitor (Figure S13, Supporting Information) exhibits energy densities ranging from 0.027 to 3.25 µWh cm^−2^ at power densities ranging from 1.93 to 0.45 mW cm^−2^, which are comparable to other carbon‐based supercapacitors, including 2D‐planar, 3D‐printed, and laser‐manufactured. As the scan rate is increased, a decrease in capacitance can be observed (Figure 4d). One of the notable advantages of the laser‐upcycled GA supercapacitors is their ability to retain a relatively high capacitive behavior for high scan rates (≈25.3 F cm^−2^ at 400 mV s^−1^) even for considerably thicker electrodes (≈3 mm) compared to typical supercapacitor electrodes (<0.1 mm), attributable to the laser‐engineered periodically‐oriented 3D networks facilitating ion diffusion.^[^ ^14^, ^15^ ^]^ Moreover, the 3D‐printed supercapacitor exhibits excellent cycle stability, with a less than 10% variation (≈94% retention) observed after 10 000 cycles at 1 A g^−1^ (Figure 4d), indicating clear implications for application in energy storage. However, from the insets of Figure 4d, a substantial voltage drop can be observed. The Nyquist plots obtained from electrochemical impedance spectroscopy (EIS) indicate that the electrode resistance is ≈20 ohms (Figure S14, Supporting Information), significantly higher than that measured from the initial electrical resistance measurements conducted for the surface (≈6.1 ohms, Figure 2h). This may be attributable to the inhomogeneity of the macrostructures along the *z*‐axis, commonly reported for carbon‐based structures formed by laser irradiation due to the induced thermal gradient.^[^ ^30^, ^59^ ^]^

## 3 Conclusion

In conclusion, we developed a PBF‐inspired AM technique capable of the direct preparation of architecture‐engineered GAs through the localized graphitization of hemoglobin, and further indicated the applicability of the laser‐upcycled GAs as electrodes of high‐performance 3D supercapacitors. The laser‐induced conversion of hemoglobin into GA was reflected in the morphological evolutions, increase in electrical conductivity, and decrease in volumetric density with an increase in energy density. The laser‐upcycled GA possessed a periodically oriented network that corresponded to the laser‐scanned path, indicating the architecture‐engineering capabilities of the laser‐based AM technique. Moreover, the network walls of the laser‐upcycled GA were uniquely decorated with fibrous graphitic structures, which can significantly improve the surface area. The surface‐decorated morphology was attributable to catalyzed graphitization by the iron‐based nanoparticles formed due to the inherent iron content of hemoglobin. Typically for the synthesis of nanostructure‐decorated graphene macrostructures, the addition of valuable pre‐synthesized carbon nanomaterials is required.^[^ ^60^, ^61^, ^62^ ^]^ By contrast, in this study it was shown that the laser‐induced graphitization of hemoglobin, sourced from commodity animal blood biowaste, can directly form nanostructure‐decorated graphene macrostructures without additives. Furthermore, proof‐of‐concept demonstrations indicated that the laser‐upcycled GAs can be successfully applied toward energy storage applications, particularly as the electrodes of symmetric supercapacitors. The 3D‐supercapacitors fabricated in this study using the hemoglobin‐derived laser‐upcycled GAs exhibited exceptional device performances, including high specific‐capacitance (≈54.9 F g^−1^), high‐rate capabilities, and excellent cycle‐stability (≈94% retention), despite the large electrode‐thickness, attributable to the laser‐engineered 3D‐architecture facilitating ion diffusion. In the future, by optimizing process parameters, such as laser parameters, scanning schemes, architecture dimensions, and precursors, it can be expected that various aspects can be tuned, including printing resolution, material composition, and structural morphology particular for the intended application, offering high process customizability. Not only does this study introduce a new facile and chemical‐free AM approach to 3D‐print GAs with engineered architectures, but also provides the necessary proof‐of‐concept of utilizing lasers to upcycle and upgrade biowastes toward the 3D printing of high‐value functional materials for future advanced applications and carbon‐based product development.

## 4 Experimental Section

### Laser Printing

Bovine‐derived freeze‐dried hemoglobin (MP Biomedical, USA) was used as the feedstock precursor material. Prior to irradiation experiments, the as‐purchased hemoglobin flakes (millimeter size) were grounded into powder form (micrometer size) using a mortar and pestle (Figure S15, Supporting Information). A YLR‐100 laser system (IPG Photonics, USA) which generated a continuous laser beam with a central wavelength of ≈1060 nm was used for the printing process. All irradiation experiments were conducted in a windowed gas‐flow chamber with an N_2_ input and a vacuum output gas line (Figure S16, Supporting Information). The N_2_ gas prevents unwanted oxidation during laser irradiation, which can lead to severe structural damage (i.e., combustion). The vacuum line provides a safe route to extract unwanted gaseous byproducts that formed during the irradiation procedure. The laser beam was scanned across a bed of hemoglobin powder (bed thickness: ≈1 cm) using a Focus Shifter digital galvanometer laser scanner system (Raylase GmbH, Germany). The diameter of the laser beam on the powder bed surface was measured to be ≈500 µm. With a constant beam diameter *ω*, the power *P* and scanning speed *v* of the laser beam were adjusted from 1 to 14 W, and 4 to 0.25 mm s^−1^, respectively. The resulting laser energy density *ED* for each power‐scanning speed combination was determined according to Equation 1. The laser processing conditions for each structure in this study are mentioned according to the determined energy density value.

(1) $$ED=\frac{P}{\omega v}\;$$

For all experiments from Section 2.2 and onwards, square macrostructures were printed by raster scanning the laser beam with a hatch spacing of 400 µm over a 1 cm by 1 cm area. As the line structures formed by a single scan for the energy densities investigated in this study were all greater than 400 µm (Figure 1d), successive raster scans scanned over a pre‐existing structure resulting in a multi‐scanning scheme. Such multi‐scanning schemes have shown benefits in progressing graphitization and improving electrical conductivity of the overall structure.^[^ ^59^ ^]^

### Material Characterizations

Width *w*, length *l*, and height *h* measurements were conducted using a VK‐X3050 confocal laser scanning microscope (Keyence, Japan). Mass *m* measurements were conducted using an HRB‐203 electronic precision scale (Tree, USA). Volumetric densities *D_V_* were calculated using values obtained by dimensional and mass measurements according to Equation 2.

(2) $$D_{V}=\frac{m}{w\times l\times h}\;$$

SEM images were obtained using an Inspect F50 scanning electron microscope (Thermo Fisher Scientific, USA). Prior to SEM observations, iridium coatings with a thickness of ≈10 nm were applied by ion sputtering. SEM‐based elemental analyses were conducted in conjunction with SEM observations using an X‐MAX energy dispersive X‐ray spectroscope (Oxford Instruments, UK). TEM images were obtained using a Talos F200X transmission electron microscope (Thermo Fisher Scientific, USA). Prior to TEM observations, the laser‐printed macrostructures were grinded into powder form and dispersed in an ethanol solution. The prepared sample‐containing solution was dropped onto a TEM grid and air‐dried before observations. TEM‐based elemental analyses were conducted in conjunction with TEM observations. Raman spectra were obtained using a LabRAM Aramis laser‐excited Raman spectrometer (Horiba, Japan). The excitation wavelength and laser power for Raman analyses were set to 532 nm and ≈36 mW, respectively. The electrical resistance measurements were conducted using a 101 basic digital multimeter (Fluke, USA) by the two‐probe method.

### Energy Storage Device Fabrication

Pairs of identical square macrostructures were printed by raster scanning the laser beam in an area of 1 cm by 1 cm with a hatch spacing of 400 µm. The as‐printed macrostructures were further cleaned of residual debris by stirring in deionized water for 30 min and subsequently drying in an 80 °C oven for 4 h. After drying, any additional residual debris adhering to the material was removed. Glass microfiber filter papers were cut into desired geometries and subsequently soaked in 5 m H_2_SO_4_ electrolyte solution for 24 h. To assemble the supercapacitor (Figure S12, Supporting Information), an electrolyte‐infused filter was carefully placed over two identical square macrostructures each with an overlap of 40% to bridge the two macrostructures. A glass slab was placed on top of the bridging filter paper as a weight to ensure constant contact between the electrolyte and the macrostructure as well as to prevent electrolyte runoff and water evaporation. Electrochemical characterizations were performed by directly contacting the working and counter electrodes to the edges of the macrostructure where the filter paper is not overlapping.

### Electrochemical Characterizations

CV and CP curves were obtained using an Interface 1000 potentiostat (Gamry Instruments Inc., USA). CV curves were obtained for scan rates of 200, 100, 80, 60, 40, 20, 10, and 5 mV s^−1^, and CP curves were obtained for current densities of 1.4, 1.0, 0.7, 0.3, and 1.0 A g^−1^. Absolute capacitance *C_A_* was calculated using the CV curve according to Equation 3.

(3) $$C_{A}=\frac{\int_{V_{i}}^{V_{f}}I\left(V\right)\;dV}{2\times v\times \left(V_{f}-V_{i}\right)}\;$$

where *v* is the scan rate, *V_f_* and *V_i_* are the potential limits of the CV curves, and *I(V)* is the voltametric current. The contact area between the filter paper and one macrostructure was considered to be ≈0.4 cm^2^ (≈0.8 cm^2^ for two electrodes). With an electrode thickness of ≈0.3 cm, the active volume per macrostructure was considered to be ≈0.12 cm^3^ (≈0.24 cm^3^ for two electrodes). The active mass per macrostructure was considered to be ≈0.0035 g (≈0.007 g for two electrodes), which was calculated by the volume and volumetric density obtained in Figure 2g, (volumetric density of ≈0.03 g cm^−3^). The specific areal, volumetric, and gravimetric capacitances were calculated by dividing the absolute capacitance by the contact area, active volume, or active mass, respectively. CV and CP curves were obtained for 4 charge‐discharge cycles, and the 4th cycle was plotted. Prior to cycle stability measurements, the fabricated supercapacitors were initially cycled for 500 times at a current density of 1.0 A g^−1^ to stabilize the device. Ragone plots were prepared by calculating the areal supercapacitor energy density and power density using the CP curves obtained at different current densities. Energy density *E_SC_* was calculated according to Equation 4.

(4) $$E_{SC}=\frac{1}{3600\;\times \;A}\;\int_{t_{i}}^{t_{f}}iV_{o}dt$$

where *A* is the contact area (≈0.8 cm^2^ for two electrodes), *i* is the discharge current density, *V_o_* is the discharge potential window after the voltage drop, and *t_i_* to *t_f_* is the discharge time. Moreover, the power density *P_SC_* was calculated according to Equation 5.

(5) $$P_{SC}=3600\times \frac{E_{SC}}{t_{f}-t_{i}}$$

EIS measurements were conducted with an alternating current with a root mean square of 0.0001 A, for a frequency range of 0.1 to 1 000 000 Hz (1MHz). Nyquist plots were obtained from the obtained EIS measurements.

## Conflict of Interest

The authors declare no conflict of interest.

## Supporting information



Supporting Information

## Data Availability

The data that support the findings of this study are available from the corresponding author upon reasonable request.
