# Supplementary material for: Laser Upcycling of Hemoglobin Protein Biowaste into Engineered Graphene Aerogel Architectures for 3D Supercapacitors
Source: Adv Sci (Weinh). 2024 Dec 31;12(8):2412588. doi: 10.1002/advs.202412588 (PMC11848628; doi:10.1002/advs.202412588)
Supplement: Supplementary file 1 — Supporting Information [file ADVS-12-2412588-s001.pdf]

ADVANCED SCIENCE
Open Access

Supporting Information

for *Adv. Sci.*, DOI 10.1002/advs.202412588

Laser Upcycling of Hemoglobin Protein Biowaste into Engineered Graphene Aerogel Architectures for 3D Supercapacitors

*Shuichiro Hayashi, Marco Rupp, Jason X. Liu, Joseph W. Stiles, Ankit Das, Amelia Sanchirico, Samuel Moore and Craig B. Arnold**

Supporting Information

Laser Upcycling of Hemoglobin Protein Biowaste into Engineered Graphene Aerogel Architectures for 3D Supercapacitors

*Shuichiro Hayashi, Marco Rupp, Jason X. Liu, Joseph W. Stiles, Ankit Das, Amelia Sanchirico, Samuel Moore, and Craig B. Arnold**

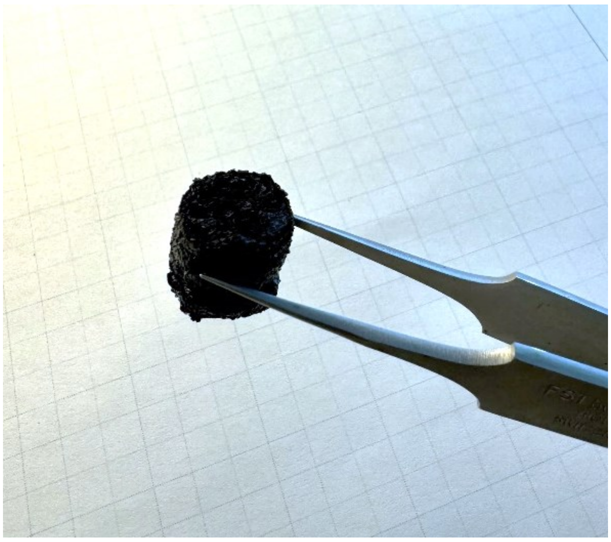

Figure S1. Photograph of the 3D-printed graphene aerogel macrostructure. The printed macrostructure is mechanically rigid, and can be easily picked up and handled without crumbling.

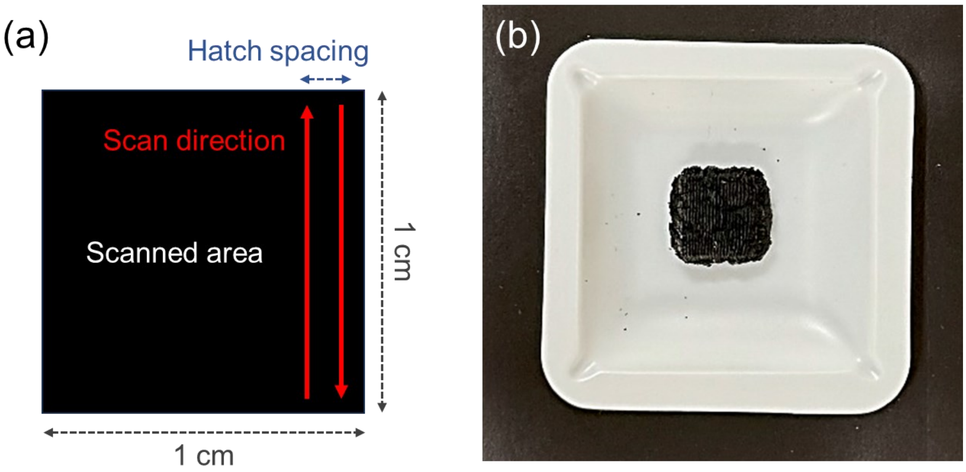

Figure S2. (a) Schematic illustration of the raster scanning process. Hatch spacing was set to be 400 μm. (b) Photograph of the square macrostructure laser-printed with an energy density of 24 J mm^{-2}.

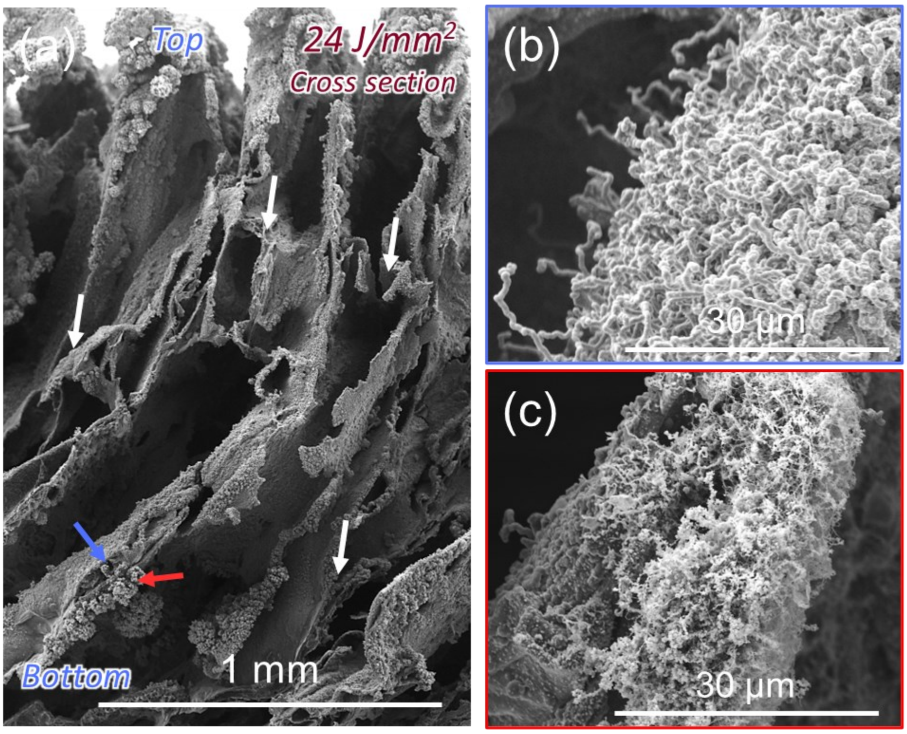

Figure S3. (a) Cross-sectional SEM image of a macrostructure printed with an energy density of 24 J mm^{-2}. White arrows indicate regions where neighboring cells are bridged. The macrostructure was cut perpendicularly to the raster scanning direction. (b) and (c) Higher magnification SEM images of the regions indicated with the blue and red arrows in (a), respectively.

ED [J/mm^2]	I_D/I_G	$FWHM_{2D}$
1	N/A	N/A
2	N/A	N/A
4	1.11	N/A
6	0.82	N/A
9	0.53	72
16	0.29	62
24	0.15	51

Figure S4. I_D/I_G ratios and full widths at half maximums of the 2D peak ($FWHM_{2D}$) calculated from the Raman spectra of each energy density (*ED*) shown in Figure 2f. For the spectra where a distinctive D, G, or 2D peak was not observed, an I_D/I_G and/or $FWHM_{2D}$ value cannot be calculated and thus denoted as N/A. A smaller I_D/I_G and $FWHM_{2D}$ value indicates a higher crystallinity of graphitic carbon.

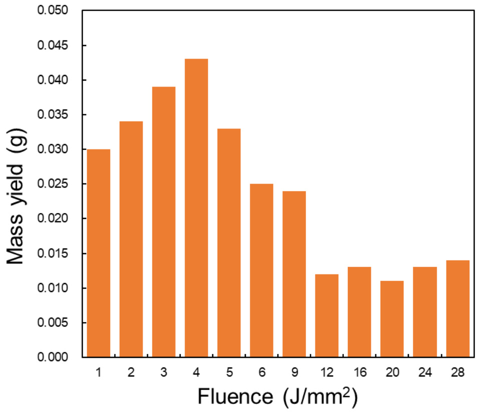

Figure S5. Measured mass yield of macrostructures printed with different energy densities.

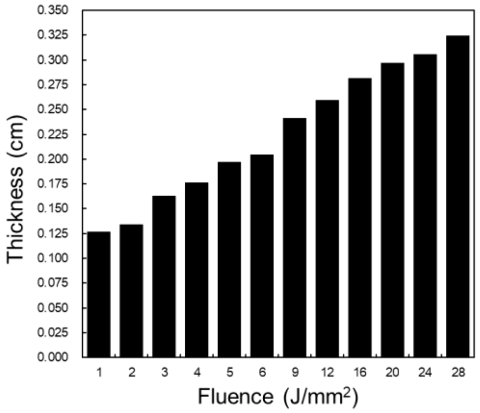

Figure S6. Measured thicknesses (z-axis) of macrostructures printed with different energy densities.

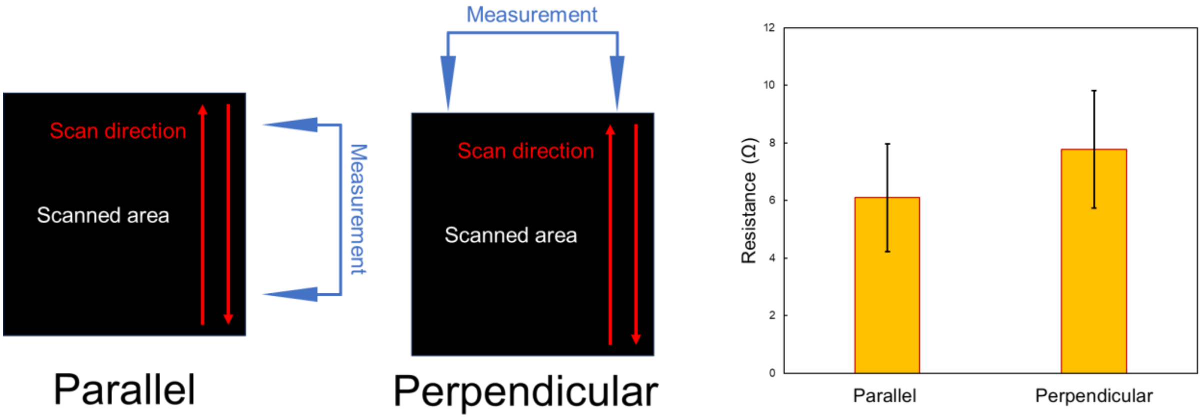

Figure S7. Electrical resistances of the same macrostructure printed with an energy density of 24 J mm^{-2}, measured for a distance of ~8 mm with different probe configurations. The error bars indicate the standard deviation for a sample size of n=5.

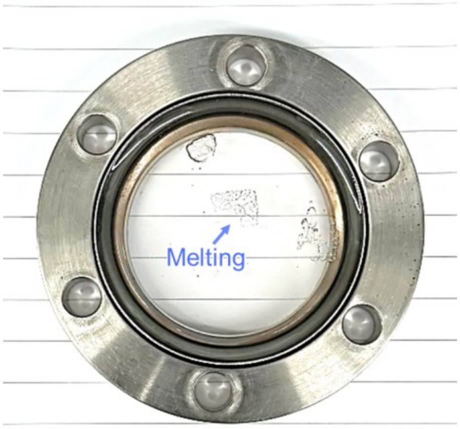

Figure S8. Optical glass window of the reaction chamber. Evident melting of the glass window was observed for excessively high energy densities.

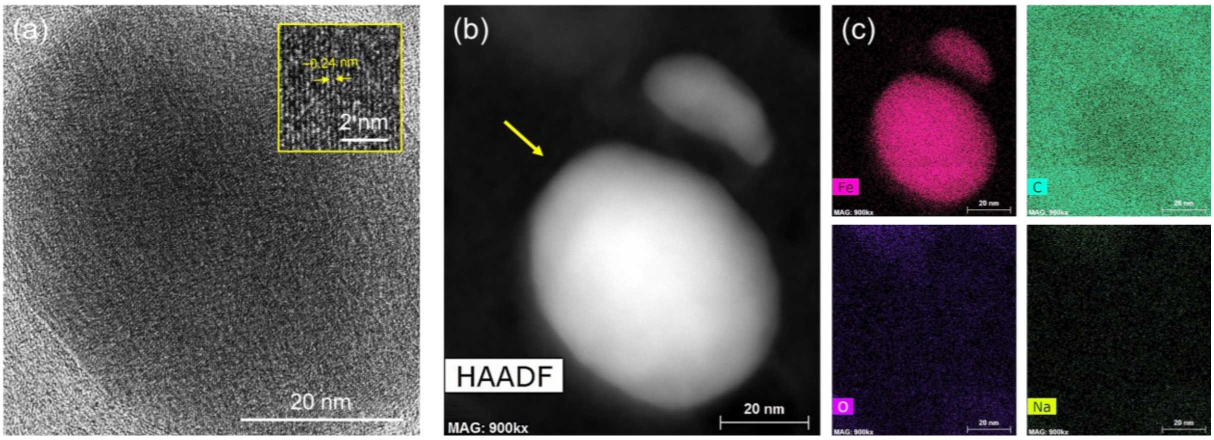

Figure S9. (a) TEM image of a dense nanoparticle exhibiting lattice fringes with a spacing of ~0.24 nm. (b) High-angle annular dark-field (HAADF) image of the field-of-view analyzed by energy dispersive X-ray spectroscopy. The nanoparticle observed in (a) is indicated with the yellow arrow in (b). (c) Obtained elemental maps of iron [Fe], carbon [C], oxygen [O], and sodium [Na] for the field-of-view. The elemental map of Na indicate that the observed nanoparticles are not salt-based crystallites as previously reported for hemoglobin-derived materials.

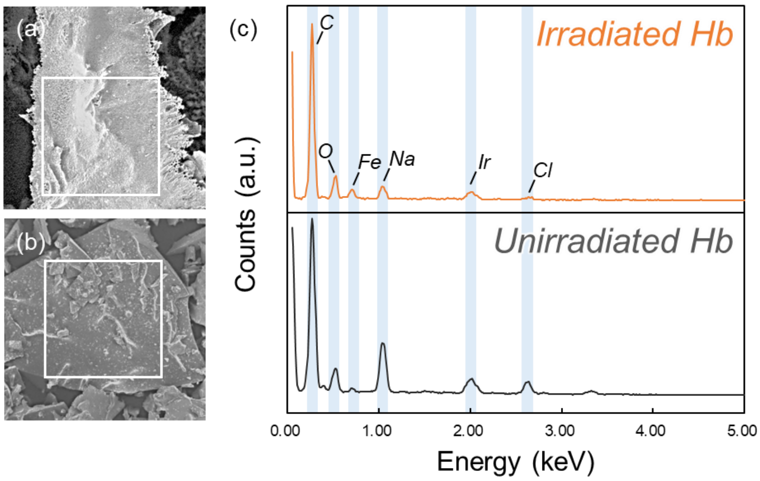

Figure S10. (a) and (b) SEM images of the analyzed regions for irradiated hemoglobin (i.e., macrostructure printed with an energy density of 24 J mm^{-2}) and unirradiated hemoglobin (i.e., as-purchased powder), respectively. (c) EDS spectra obtained from each of the respective regions.

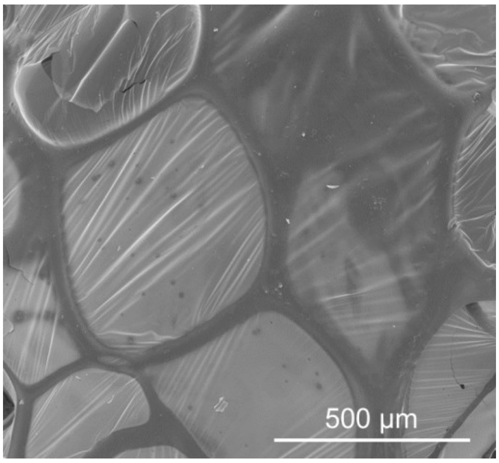

Figure S11. SEM image of the surface of a graphene aerogel prepared from powdered hemoglobin via furnace-assisted graphitization, according to the methodology mentioned in "S. Ozden, et al., *Materials Today* 2022, 59, 46".

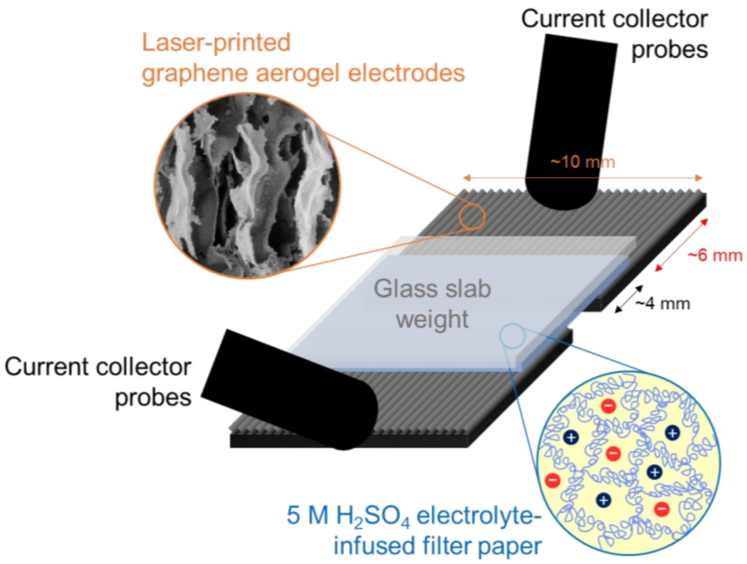

Figure S12. Schematic illustration of the supercapacitor characterization setup. An electrolyte-infused glass fiber filter paper was placed over two identical 1 cm by 1 cm square macrostructures with a 40% overlap to bridge the two macrostructures. A glass slab was further placed over the filter paper as a weight, and to prevent electrolyte run off. For measurements, probes were directly contacted with the uncovered macrostructure.

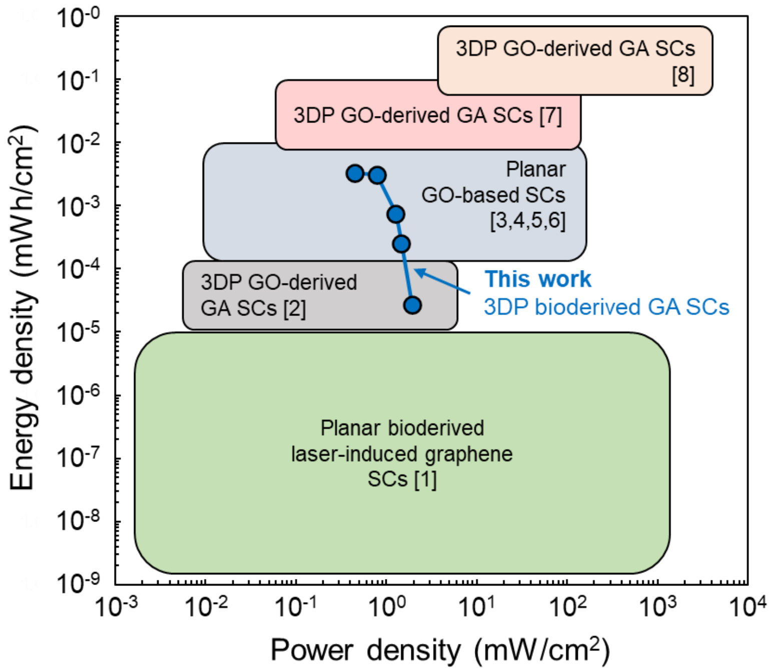

Figure S13. Ragone plot of the supercapacitor (SC) fabricated in this study, with other reported values for comparison. Performances of other SCs are indicated as a broadly-defined range, extracted from previously reported Ragone plots. 3DP: 3D-printed, GO: graphene oxide, GA: graphene aerogel.

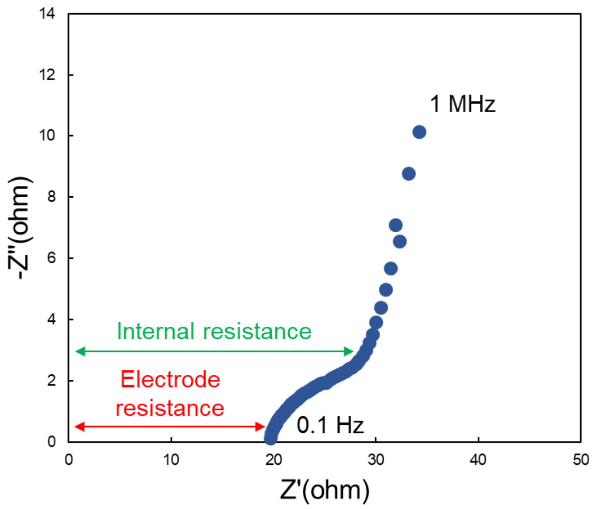

Figure S14. Nyquist plot of the symmetrical supercapacitor fabricated using laser-printed macrostructures obtained from EIS measurements for a frequency range of 0.1 Hz to 1 MHz. The plot indicates an electrode resistance of ~20 ohms (region indicated with red), and an total device internal resistance of ~30 ohms, which includes various elements such as the electrolyte resistance (region indicated with green).

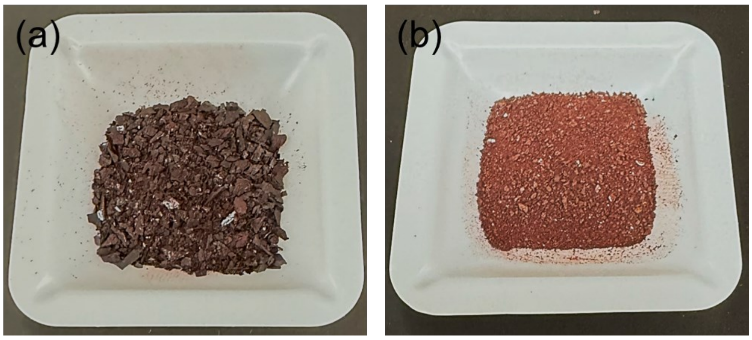

Figure S15. Photographs of the (a) as-purchased and (b) ground up freeze-dried hemoglobin powder.

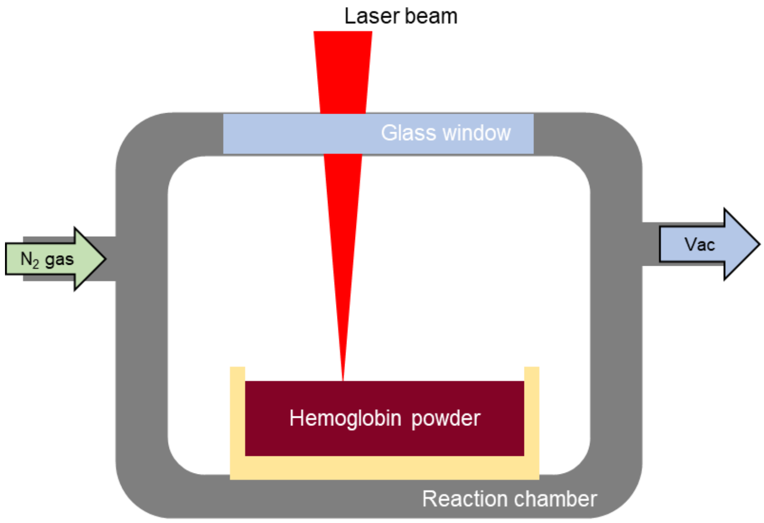

Figure S16. Schematic illustration of the laser irradiation experimental setup. N_2 gas was flown into the reaction chamber and vacuum (Vac) was pulled out while laser irradiation. The N_2 gas prevents unwanted oxidation during laser irradiation, which can lead to severe structural damage (i.e., combustion). The vacuum line provides a safe route to extract unwanted gaseous byproducts that formed during the irradiation procedure.

References

1. A. C. Bressi, A. Dallinger, Y. Steksova, F. Greco, *ACS Appl Mater Interfaces* **2023**, *15*, 35788.
2. S. Yuan, W. Fan, D. Wang, L. Zhang, Y. E. Miao, F. Lai, T. Liu, *J Mater Chem A Mater* **2021**, *9*, 423.
3. G. Qu, J. Cheng, X. Li, D. Yuan, P. Chen, X. Chen, B. Wang, H. A. Peng, *Adv. Mater.* **2016**, *28*, 3646–3652.
4. Z. Xiong, C. Liao, W. Han, X. Wang, *Adv. Mater.* **2015**, *27*, 4469–4475.
5. Y. Xu, Z. Lin, X. Huang, Y. Liu, Y. Huang, X. Duan, *ACS Nano* **2013**, *7*, 4042–4049.
6. Y. Shao, M. F. El-Kady, C.-W. Lin, G. Zhu, K. L. Marsh, J. Y. Hwang, Q. Zhang, Y. Li, H. Wang, R. B. Kaner, *Adv. Mater.* **2016**, *28*, 6719.
7. X. Tang, H. Zhou, Z. Cai, D. Cheng, P. He, P. Xie, D. Zhang, T. Fan, *ACS Nano* **2018**, *12*, 3502.
8. C. Zhu, T. Liu, F. Qian, T. Y. J. Han, E. B. Duoss, J. D. Kuntz, C. M. Spadaccini, M. A. Worsley, Y. Li, *Nano Lett* **2016**, *16*, 3448.
